# Biological Sex Is Binary and Rooted in Anisogamy

**DOI:** 10.1111/ele.70426

**Published:** 2026-07-10

**Authors:** Tim Janicke, Göran Arnqvist, Pierre‐André Crochet, Patrice David, Philippe Jarne, Jussi Lehtonen, Thomas Lenormand, Edward H. Morrow, Geoff A. Parker, Paul A. Saunders, Jeanne Tonnabel, Frédéric Veyrunes, Christoph R. Haag

**Affiliations:** ^1^ CEFE, Univ Montpellier, CNRS, EPHE, IRD Montpellier France; ^2^ Department of Ecology and Genetics Uppsala University Uppsala Sweden; ^3^ Department of Biological and Environmental Science University of Jyväskylä Jyväskylä Finland; ^4^ Department of Environmental and Life Sciences Karlstad University Karlstad Sweden; ^5^ Department of Evolution, Ecology and Behaviour University of Liverpool Liverpool UK; ^6^ ISEM, Univ Montpellier, CNRS, IRD Montpellier France

**Keywords:** definition, gametes, sexual dimorphism, sexual reproduction

## Abstract

The definition of biological sex has become a renewed focus of societal debate, fuelled by the conflation of biological principles with discussions of human gender diversity. Here, we argue that conceptual clarity critically depends on separating these domains. Drawing on evolutionary theory and empirical evidence, we maintain that biological sex is best defined as a binary classification of male and female reproductive strategies rooted in anisogamy, characterised by the production of two discrete gamete types of different sizes. We stress that gamete size constitutes the ultimate criterion for biological sex and that this definition applies consistently across sexual systems, from separate‐sexed species to hermaphrodites, irrespective of variation in karyotype, hormonal profile, somatic phenotype, or behaviour. Further, we emphasise that evolutionary insights offer a coherent explanation for recurring, though not universal, associations between biological sex and patterns of sex‐specific selection, sexual dimorphism and parental care. We conclude that the definition of biological sex as a binary classification based on gamete size is a powerful scientific framework compatible with the diversity of sexual phenotypes found in anisogamous organisms and distinct from the concept of human gender.

## Graphical abstract

Drawing on empirical evidence and evolutionary theory, we argue that biological sex is most coherently defined as a binary classification of reproductive strategies rooted in anisogamy (the production of large or small gametes). This framework accommodates the diversity of sexual phenotypes while maintaining a clear distinction between biological sex and human gender.

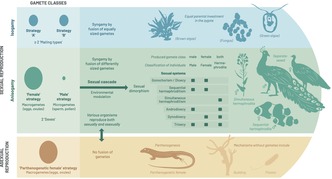

## 1 Introduction

Researchers across disciplines have long been confronted with a societal debate about the concept of biological sex that has resurfaced with renewed intensity in recent years (Griffiths 2020; DiMarco et al. 2022; Coyne and Maroja 2023; McLaughlin et al. 2023; Rehmann‐Sutter et al. 2023; Arnold et al. 2024; Velocci 2024; Eppley et al. 2026). This controversy stems largely from efforts to reconcile scientific insights and facts in biology with human societal values—an endeavour predestined to misunderstandings and cross‐disciplinary tensions. Biology, as a scientific discipline, aims to describe the complexity of life and to understand the mechanisms that generate and govern this diversity. It is not designed to prescribe ethical norms or moral values in human societies, which fall within the domain of other disciplines. Conversely, contemporary societal values or political agendas should not guide the interpretation of empirical observations in biology or the conceptualisation of life's complexity. Doing so constitutes a ‘reverse naturalistic fallacy’ (or ‘moralistic fallacy’): the illogical attempt to derive an ‘is’ from an ‘ought’, thereby conflating the empirical with the normative (Davis 1978). Based on these premises, we call for a more differentiated discourse on sex, one that separates the scientific conception of biological sex from societal discussions regarding the diversity of gender expressions and identities in humans.

The recognition that biological sex is rooted in anisogamy has been longstanding in biology (Minot 1888; Geddes and Thomson 1889), and there have been several recent accounts illuminating its foundations and explanatory power (e.g., Goymann et al. 2023; Hilton and Wright 2023; Griffiths and Spencer 2025; Wright 2025). Our aim here, without claiming novelty on this well‐documented topic, is to (i) explain and support the gametic definition of sex, (ii) take a stand on its most prominent criticisms and (iii) elucidate the association between biological sex and sexual dimorphism beyond anisogamy, as this issue appears to lie at the heart of recent debates.

## 2 The Gametic Definition of Biological Sex and Its Conceptual Value

Biological sex is a binary classification of reproductive strategies for propagating genetic material, defined by differences in gamete size (i.e., anisogamy). The production of large, nutritive gametes produced at a relatively low rate (i.e., macrogametes, such as eggs and ovules) is referred to as the ‘female’ strategy, whereas the production of small gametes produced at a relatively high rate (i.e., microgametes, such as sperm and pollen) is referred to as the ‘male’ strategy. Accordingly, gamete size (as an indicator of per‐gamete investment) is the ultimate criterion distinguishing male and female reproductive strategies, making the gametic definition of sex independent of any genetic or phenotypic corollaries that may be present in some systems but absent in others, such as karyotypes in species with differentiated sex chromosomes or secondary sexual traits in sexually dimorphic species. Depending on an organism's sexual system, both strategies can be mapped onto individuals (i.e., males and females in separate‐sexed organisms and sequential hermaphrodites), or within individuals as male and female sex functions (in simultaneous hermaphrodites) (Charnov 1982; Schärer 2017). In seed plants, size differences occur not at the level of the gametes themselves but at the level of the multicellular gametophytes. In these organisms, the female strategy produces megagametophytes (ovules), whereas the male strategy produces microgametophytes (pollen grains). For simplicity, and following common usage, we nevertheless refer to them as gametes here.

**FIGURE 1:**
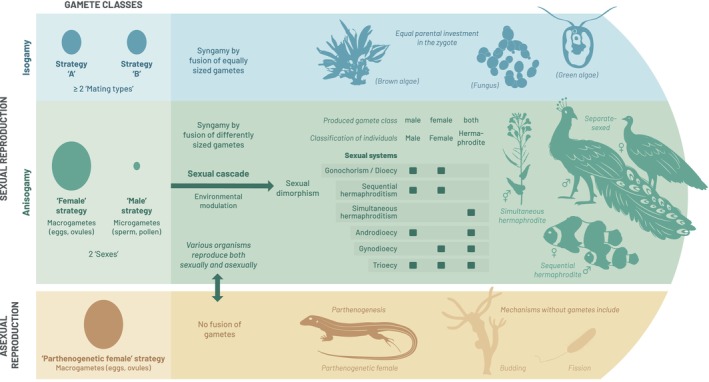
Biological sex at a glance. The binary classification of biological sex applies to anisogamous sexually reproducing organisms, in which the male strategy produces small gametes and the female strategy produces large gametes. Depending on the sexual system, one or both gametic strategies can be mapped onto individuals, making the gametic definition of sex applicable to separate‐sex organisms, sequential hermaphrodites, simultaneous hermaphrodites and mixed systems. The sexual cascade provides the theoretical framework for explaining the evolutionary drivers of sexual dimorphism. Organisms that reproduce sexually through the fusion of equally sized gametes (isogamy) do not have sexes but may possess two or more mating types. Asexual reproduction via parthenogenesis involves nutrient‐rich egg‐like gametes such that parthenogenic individuals are also often referred to as females.

The definition of biological sex based on gamete size implies that sex is defined only in anisogamous species, which produce gametes of two distinct size classes (Figure 1), but not in isogamous species or species in which reproduction does not involve gametes. It therefore mostly pertains to sexually reproducing organisms, although asexual individuals producing large gametes are sometimes still referred to as ‘females’. Isogamous species, including some fungi and algae, do not have sexes but may possess distinct mating types (sometimes more than two; Lehtonen, Kokko, et al. 2016). Even though anisogamy has multiple evolutionary origins (Kirk 2006; Umen and Coelho 2019), it occurs in the vast majority of sexually reproducing eukaryotes covering some fungi and nearly all multicellular plants and animals. Importantly, evolutionary theory predicts that selection favours the evolution of exactly two, and not more than two, gametic strategies (Lehtonen and Parker 2014)—a prediction that is strongly supported across anisogamous species: with the exception of a few rare lineages showing slightly overlapping yet bimodal gamete‐size distributions (e.g., in certain algae; Clifton and Clifton 1999), all anisogamous species exhibit two distinct, non‐overlapping size classes of gametes. Thus, despite independently replicated evolutionary origins, the emergence of anisogamy is associated with only two classes of gametes, and never more. It is precisely this highly consistent occurrence of two distinct gamete size classes across anisogamous species that renders the definition of biological sex based on gamete size inherently binary. However, defining biological sex as binary does not imply that individuals necessarily fall into two classes, as they can produce male, female, both, or neither gamete class.

The term ‘anisogamy’, which strictly refers to the presence of ‘unequal’ gametes, is most often conceptualised in terms of differences in their size. In fact, the theoretical foundations for the evolution of anisogamy centre on disruptive selection acting on pre‐zygotic investment per gamete on the one hand and on gamete number on the other hand, whereby increased provisioning can be achieved only at the cost of producing fewer gametes at a given time (Kalmus 1932; Parker et al. 1972; Bulmer and Parker 2002). Parental provisioning of resources to the zygote is assumed to require the physical packing of resources, such that greater pre‐zygotic investment results in gametes of larger volume and therefore larger size.

Another point worth clarifying concerns the biological sex of non‐reproductive individuals. Male and female strategies can be mapped onto these individuals according to the developmental trajectories they possess (Griffiths and Spencer 2025). Therefore, an individual producing one of the two gamete types during its lifetime can be associated with the corresponding strategy and classified as a male or a female, whereas an individual producing both types during its lifetime can be classified as a sequential (i.e., male or female at different times) or simultaneous hermaphrodite (i.e., simultaneously having male and female sex functions). This approach allows assigning male and female strategies to, for example, sexually inactive juveniles, post‐reproductive adults, or organisms exhibiting seasonal variation in gamete production. However, it is not always meaningful to assign a sex to every individual, as in the case of early embryos of species with environmental sex determination, sequential hermaphrodites in the process of switching sex (Griffiths 2020), or diploid individuals in the life cycle of mosses and liverworts (Bachtrog et al. 2014; Coelho et al. 2018).

It is also worth mentioning that the gametic definition of biological sex is nothing more than a classification of reproductive strategies. These strategies clearly dominate multicellular life and happen to be historically termed ‘male’ and ‘female’ but they could just as well be called the ‘microgamete‐producing’ strategy and the ‘macrogamete‐producing’ strategy. From this perspective, the debate is in part terminological, and replacing the long‐established terms ‘male’ and ‘female’ seems counterproductive. Importantly, defining sex by gamete size does not imply universal differences in any traits other than the type of gamete produced. Whether the male and female strategies are associated with morphological, physiological, or behavioural traits beyond the dimorphism in gamete size is an important question (see Box 1), but this issue is independent of the definition of biological sex.

BOX 1 **Sex Differences Arising From Anisogamy.**

In light of the most common criticisms of defining biological sex by gamete size, the question arises whether anisogamy has explanatory value. When Darwin (1859, 1871) founded the discipline of evolutionary biology, he was particularly intrigued by sex differences. His assignment of males as primarily competing for mates, whereas females being more often choosy about whom they mate with, was later taken up by Bateman (1948), who hypothesised that this sex difference in the operation of sexual selection is caused by higher reproductive benefits of accessing multiple mates for males than for females. Importantly, Bateman already speculated that Darwin's postulated sex difference in sexual selection is ultimately rooted in anisogamy. These insights were later synthesised in the so‐called ‘Darwin–Bateman Paradigm’, which constitutes a central pillar of modern evolutionary biology for explaining the divergence of reproductive strategies grounded in gametic sex (Dewsbury 2005; Janicke 2024a). Bateman's idea was inspired by data he obtained from experimental work on fruit flies, which was pioneering for its time but later subject to severe criticism regarding statistical analysis and experimental design (Snyder and Gowaty 2007; Gowaty et al. 2012; Hoquet et al. 2020). These criticisms continue to be used as key arguments, even in popular science books (Cooke 2022; Fuentes 2025; Kamath and Packer 2025), to dispute the view that sex differences have an evolutionary basis rooted in anisogamy. Yet, the scientific value of Bateman's theoretical conceptualisation of the evolution of sex differences does not hinge on the robustness of one particular empirical dataset, as these merely served as a catalyst for his conceptual reasoning (Morimoto 2020). Importantly, critics of Bateman's work often seem to overlook the fact that repetitions of his experiment with the same fruit fly system, designed to avoid the potential pitfalls he faced, consistently support all the conclusions he drew from his data (e.g., Bjork and Pitnick 2006; Davies et al. 2023), and that his main findings have been verified across many other taxa (Janicke et al. 2016).

Darwin's and Bateman's foundational work has been subject to constant theoretical refinements and extensions. Parker's *sexual cascade* outlines the evolutionary sequence following the emergence of anisogamy: in external fertilisers, competition among male gametes led to the evolution of greater mobility and intensified pre‐copulatory sexual selection, which on land became associated with internal fertilisation and the expansion of Darwinian sex roles (Parker 2014). Formal theoretical models have confirmed that anisogamy can trigger a divergence in reproductive strategies: the production of excess gametes by the male strategy makes it more prone to benefit from additional matings (Lehtonen 2022; Lehtonen and Parker 2024) and to invest more in competition than the female strategy (Lehtonen, Parker, et al. 2016), which in turn affects sex differences in parental care (Fromhage and Jennions 2016). Empirical data strongly support these theoretical predictions. Across diverse animal taxa, the male strategy, defined by gamete size, typically gains more from additional matings than the female strategy, a difference associated with the evolution of more elaborate male secondary sexual traits and female‐biased parental care (Janicke et al. 2016).

Despite the prevailing patterns described above, evolutionary theory also predicts that sex differences in sexual selection can vary (e.g., Lehtonen 2022; Lehtonen et al. 2024; for conditions under which sex‐specific selection can reverse) and that sex‐specific phenotypes can evolve independently of such differences through ecological character displacement (De Lisle 2019; Li and Kokko 2021). Moreover, females have been found to commonly benefit from having multiple partners (Arnqvist and Nilsson 2000; Fromonteil et al. 2023) and in some species this leads them to compete more for mates and invest less in care than males (Hare and Simmons 2019; Fritzsche et al. 2021)—a phenomenon already acknowledged by Darwin (1871). Emblematic examples of species in which ornamented females compete for choosier males include seahorses and shorebirds such as phalaropes and painted snipes (Jones and Avise 2001; Janicke 2024b). Finally, environmental conditions are known to alter sex differences in sexual selection imposed by gamete size leading to flexible sex roles even within species (Cornwallis and Uller 2010; Miller and Svensson 2014; Garcia‐Roa et al. 2020).

The outlined secondary modifications indicate that the evolutionary forces driving sex differences beyond anisogamy are diverse and complex. However, they do not refute the theoretical work and empirical evidence showing that anisogamy, as the ultimate defining criterion of biological sex, explains a significant fraction of the diversity in sexual phenotypes we can observe in nature. Empirical observations from across the eukaryotic tree of life are consistent with the predictions of the sexual cascade, of which the Darwin–Bateman paradigm is one important building block. In this sense, beyond the binary definition of biological sex by gamete size, little may appear to be universally binary. However, there are well‐documented associations between anisogamy and various forms of sexual dimorphism in most lineages, making the definition of biological sex by gamete size a useful starting point for studying the evolutionary origins of phenotypic diversity.

Finally, we should highlight why the biological definition of sex based on gamete size is useful. There are at least three interrelated reasons: First, the evolution of anisogamy from isogamy represents one of the earliest intra‐specific differentiations of reproductive strategies in sexually reproducing eukaryotic lineages. Second, anisogamy is evolutionarily stable and, together with the previous point, is therefore very widespread across the eukaryotic tree of life. Thus, the two gametic strategies can be distinguished across a very broad taxonomic range, which stands in stark contrast to any other biological trait that is sometimes used to assign sex (e.g., not all organisms have sex chromosomes, sex hormones, ornaments, genitalia, courtship behaviour). Third, gamete size dimorphism has far‐reaching evolutionary consequences that help us understand the diversity of lifeforms in nature.

## 3 Robustness of the Gametic Definition Across Sexual Systems

A number of criticisms have been raised regarding the definition of biological sex in terms of gamete size (Table S1). Presumably the most frequent concern is that binary sex based on gamete size does not account for the whole biological diversity of sexual phenotypes beyond anisogamy. Proponents of pluralistic approaches argue that sex is not binary because sex‐related characteristics are too complex to be confined to gamete size (McLaughlin et al. 2023; Smiley et al. 2024; Velocci 2024). As mentioned above, defining biological sex by gamete size does not in itself imply any universal relationship with other morphological, physiological, genetic, behavioural, or life‐history traits. However, a very long‐standing and continuously refined body of evolutionary theory predicts that anisogamy is indeed a precursor that can, under a certain set of conditions, lead to further divergence of reproductive strategies (Schärer et al. 2012). As we explain in Box 1, this framework (referred to as the ‘sexual cascade’; Parker 2014) offers a powerful toolkit for predicting large‐scale tendencies in sex differences while acknowledging their diversity.

Other objections relate to particular sexual systems, life histories or rare species‐specific character states, such as simultaneous hermaphrodites, individuals unable to produce gametes, species with giant sperm, or cases in which gamete production is restricted to certain life stages. Yet, in all these cases the gametic definition remains applicable if sex is interpreted as a reproductive strategy rather than a fixed property of individuals (see Table S1 for detailed clarifications of individual critiques). Alternative definitions face substantial difficulties. For instance, the multivariate model of sex attempts to incorporate multiple binary and non‐binary traits to describe the sexual phenotype (McLaughlin et al. 2023), but it is unclear how different traits should be weighted and combined in a way that is general, biologically meaningful, and grounded in evolutionary theory. Given that many proposed traits, such as morphology, behaviour and levels of sex hormones, are expressed only in certain taxa, the multivariate model precludes cross‐species comparisons and therefore does not allow us to understand the evolution of sexual strategies at broader macroevolutionary scales. Similarly, other approaches, such as the definition of sex based on the karyotype, apply only to a small set of taxa or biological contexts (where they largely coincide with the gametic definition), implicitly rely on the gametic definition (e.g., when describing traits as ‘male‐like’ or ‘female‐like’), or require accepting the existence of sperm‐producing females and egg‐producing males. These definitions tend to obscure rather than clarify the biological concepts associated with reproductive strategies and their associated traits. Even though the definition of biological sex rooted in anisogamy does not capture all variation in sexual traits, it provides a robust and widely applicable framework for understanding the evolution of reproductive strategies (Box 1).

## 4 Concluding Remarks

Up to this point, we have intentionally refrained from discussing the implications of biological sex for our understanding of sex in humans. Yet, within the outlined definitional framework, humans are not exceptional. Rather, like all other metazoans, we are anisogamous organisms, and it is this shared reproductive feature that makes the binary definition of biological sex directly applicable to humans. Because human individuals can produce only one of the two gamete types, biological sex can be mapped onto individuals, as in other species with separate sexes. Challenges can arise when applying the binary definition of biological sex in society under the assumption that biological sex is universally tied to other traits, such as karyotype, hormone levels, the morphology of primary and secondary sexual characteristics, or behaviour. This assumption, for instance, ignores the possibility that genetic variants or differences in sex development can result in a divergence between gametic sex and phenotypic correlates. Crucially, however, the definition of biological sex based on anisogamy neither relies on nor supports this assumption. Therefore, the binary definition of biological sex is fully compatible with the diversity of sexual phenotypes beyond anisogamy (Heitzmann et al. 2023; Mank 2023; Loveland et al. 2025) and provides a coherent explanation for a significant part of it. In the context of ongoing societal debates, we emphasise that defining biological sex places no constraints on the diverse spectrum of human gender identities and expressions. While biological insights can inform societal debates on sex, they do not prescribe which gender‐related traits society should assign, permit, or encourage for different sexes. Conflating human gender identities and expressions with the binary nature of biological sex is therefore not a scientific inference, but amounts to two fallacies: first, not acknowledging the diversity of human gender identities and expressions because biological sex is considered binary (‘naturalistic’ fallacy), and second, denying that biological sex is binary because human gender identities and expressions are not strictly binary (‘reverse naturalistic’ or ‘moralistic’ fallacy).

## Author Contributions

Conceptualisation: All authors. Visualisation: Tim Janicke. Project administration: Tim Janicke. Writing – original draft preparation: Tim Janicke. Writing – review and editing: All authors. Final approval: All authors.

## Funding

T.J. was funded by the CNRS and the French National Research Agency (ANR‐25‐CE02‐1229). J.L. was funded by the Research Council of Finland (grant 340130) and by the European Union (ERC, CAUSEX, 101171163). Views and opinions expressed are however those of the authors and do not necessarily reflect those of the European Union or the European Research Council. Neither the European Union nor the granting autority can be held responsible for them.

## Conflicts of Interest

The authors declare no conflicts of interest.

## Supporting information


**Table S1:** Frequently cited objections to the gametic definition of biological sex and corresponding clarifications offered by the gametic account.

## Data Availability

The authors have nothing to report.
