# Supplementary material for: Biological Sex Is Binary and Rooted in Anisogamy
Source: Ecol Lett. 2026 Jul 10;29(7):e70426. doi: 10.1111/ele.70426 (PMC13354727; doi:10.1111/ele.70426)
Supplement: Supplementary file 1 — Table S1: Frequently cited objections to the gametic definition of biological sex and corresponding clarifications offered by the gametic account. [file ELE-29-0-s001.docx]

Supplementary Material for

**Biological sex is binary and rooted in anisogamy**

Tim Janicke^1*^, Göran Arnqvist^2^, Pierre-André Crochet^1^, Patrice David^1^, Philippe Jarne^1^, Jussi Lehtonen^3^, Thomas Lenormand^1^, Edward H. Morrow^4^, Geoff A. Parker^5^, Paul A. Saunders^1^, Jeanne Tonnabel^6^, Frédéric Veyrunes^6^, Christoph R. Haag^1^

^1^ CEFE, Univ Montpellier, CNRS, EPHE, IRD, Montpellier, France.

^2^ Department of Ecology and Genetics, Uppsala University, Uppsala, Sweden.

^3^ Department of Biological and Environmental Science, University of Jyväskylä, Jyväskylä, Finland.

^4^ Department of Environmental and Life Sciences, Karlstad University, Karlstad, Sweden.

^5^ Department of Evolution, Ecology and Behaviour, University of Liverpool, Liverpool, United Kingdom.

^6^ ISEM, Univ Montpellier, CNRS, IRD, Montpellier, France.

^*^Corresponding author

[tim.janicke@cefe.cnrs.fr](mailto:tim.janicke@cefe.cnrs.fr)

This Supplementary Material includes

Table S1

#

# Supplementary Tables

**Table S1.** Frequently cited objections to the gametic definition of biological sex and corresponding clarifications offered by the gametic account.

| Critique | Reference | Clarification |
| --- | --- | --- |
| Anisogamy is decoupled from sexual dimorphism in non-gametic traits. Therefore, defining biological sex by gametes has no explanatory power. | (Dupré, 1986; Evron, 2024; McLaughlin et al., 2023; Rehmann-Sutter et al., 2023; Smiley et al., 2024; Watkins and DiMarco, 2025) | The gametic definition of biological sex does not, by itself, imply a universally valid association between anisogamy and dimorphism in non-gametic traits. Moreover, explanatory power does not require perfect correlation. In fact, anisogamy provides one of the most powerful theoretical frameworks for explaining sexual differentiation: models based on differences in gamete size predict divergent reproductive strategies, which frequently lead to differences in mating behaviour, secondary sexual traits, and parental investment. These patterns are widely supported across taxa but are general rather than universal, and much of the observed variation can itself be explained within the same evolutionary framework. Thus, anisogamy offers a predictive basis for understanding many forms of sexual differentiation, arguably more so than alternative definitions of sex that simply group traits without an underlying evolutionary rationale. |
| Not applicable to cases in which individuals produce both male and female gametes at the same time (e.g., simultaneous hermaphrodites or sporophytes of species with UV sex determination) or no gametes at all (e.g., diploid sporophytes in species with UV sex determination). | (Gorelick et al., 2013, 2017; McLaughlin et al., 2023) | This critique argues that the gametic definition is flawed because it does not always allow sex to be assigned at the individual level. The gametic definition defines sex as a reproductive strategy that can be mapped onto different biological levels, including the individual level in separate-sexed organisms and sequential hermaphrodites or sex functions in simultaneous hermaphrodites (Schärer, 2017). Other individuals have no sex, either temporarily (see main text) or permanently. In species with haploid (UV) sex determination, sex exists only in the haploid phase: female U gametophytes produce large gametes and male V gametophytes small gametes, whereas diploid sporophytes produce spores rather than gametes. The fact that some individuals lack a sex under the gametic definition reflects the biology of their life cycles rather than a limitation of the concept. |
| The gametic definition is often not operational because gamete size or other gametic properties can not always be measured due to practical limitations (e.g., in fossils). | (Eppley et al., 2026; McLaughlin et al., 2023) | This critique conflates the practical challenges of sex assignment with the conceptual definition of biological sex. In some separate-sexed species, certain individuals do not exhibit the typical phenotypic traits associated with the production of micro- or macrogametes. In such cases, assigning biological sex using proxy traits (e.g., primary or secondary sexual characters) can involve some degree of uncertainty. However, proxies approximate an underlying concept, they do not replace it. Accordingly, this is an empirical issue concerning the accuracy of sex assignment in specific contexts and does not invalidate the conceptual framework in which biological sex is defined by differences in gamete size. Variation in sexual dimorphism across or within species, as well as phenotypic variation within a given sex, does not undermine this underlying definition. |
| In some species, males produce unusually large sperm, whereas in others, female gametes are exceptionally small (e.g., ovules in orchids) making the typical distinction between sexes based on gamete size problematic. | (Gorelick et al., 2013, 2017; Watkins and DiMarco, 2025) | Cases such as giant sperm or unusually small female gametes are striking but extreme examples of the diversity found among anisogamous species. Even in these taxa the fundamental asymmetry between the two reproductive strategies usually remains intact. For example, in *Drosophila bifurca* sperm are extraordinarily long, yet their total volume remains smaller than that of eggs and they are produced at about six times the rate of egg production (Bjork and Pitnick, 2006). Likewise, in orchids the ratio of pollen to ovules produced typically exceeds 15:1 (Staedler et al., 2018). More generally, anisogamy concerns asymmetries in reproductive pre-zygotic investment. Although this is often expressed in terms of gamete size, parental investment may also occur in structures associated with the gamete. Thus, even when absolute gamete sizes appear unusual, the underlying anisogamous pattern can remain strong (e.g., in plants, maternal investment occurs largely through tissues of the female gametophyte surrounding the gamete; see main text). |
| In humans and other species, differences in sex development (DSD) can lead to a mismatch between typical genetic and phenotypic signatures of sex and can prevent some individuals from producing gametes, which makes it impossible to assign sex based on gamete size. | (DuBois and Shattuck‐Heidorn, 2021; Frederick, 2025) | Defining sex by gamete size does not imply that all individuals must fall into one of two categories. Anisogamy inherently allows at least four ways in which sex may map onto individuals: male, female, simultaneous hermaphrodite, and sex undefined. The latter refers to individuals that do not produce gametes and do not have the potential to do so. Examples include diploid UV sporophytes. It may also include individuals with DSD (Griffiths, 2020; Griffiths and Spencer, 2025; Wright, 2025). |
| Gamete production is often restricted to particular life stages (e.g., absent during juvenility, after menopause, or outside seasonal breeding periods), meaning that the gametic definition is applicable only during limited time windows within an individual’s life. | (Gorelick et al., 2013; Smiley et al., 2024) | The gametic definition of sex is fully compatible with assigning sex based on reliable proxies, even for organisms that are not currently producing gametes but have or had the potential to do so (e.g., Rifkin and Garson, 2023). |
| Anisogamy does not necessarily entail the presence of exactly two distinct gamete sizes. For instance, some algal species classified as anisogamous produce gametes that vary continuously in size. | (Watkins and DiMarco, 2025) | In a few species, gamete size may vary continuously rather than forming two clearly separated classes. Theory on the evolution of anisogamy is fully compatible with such continuous variation  (Lehtonen, 2021). In species with near-isogamy, variation in gamete size can be substantial, and although gamete size distributions remain bimodal, they may overlap as described for certain algae (e.g., Clifton and Clifton, 1999). Assigning sex to individuals can then be difficult or even impossible. However, this phenotypic variation is analogous to the size variation seen within micro- and macrogametes in fully anisogamous species (Griffiths and Spencer, 2025; Togashi et al., 2024). |
| There are no universal differences between male and female eukaryotes, neither at the diploid nor at the haploid phase of the life cycle. | (Gorelick et al., 2013, 2017) | This critique implicitly relies on the very distinction it questions: the terms ‘male’ and ‘female’ are used without an independent definition, effectively referring to the gametic categories themselves. The gametic definition of sex applies to anisogamous systems, where the two reproductive strategies are defined by the production of small versus large gametes. Claims that anisogamy itself sometimes breaks down (e.g., unusually large sperm or small eggs) typically concern extreme or ambiguous cases and do not undermine the general pattern of asymmetric reproductive per-gamete investment that characterizes anisogamy. |

# References

Bjork A, Pitnick S, 2006. Intensity of sexual selection along the anisogamy-isogamy continuum. Nature 441:742-745.

Clifton KE, Clifton LM, 1999. The phenology of sexual reproduction by green algae (Bryopsidales) on Caribbean coral reefs. Journal of Phycology 35:24-34.

DuBois LZ, Shattuck‐Heidorn H, 2021. Challenging the binary: Gender/sex and the bio‐logics of normalcy. American Journal of Human Biology 33:e23623.

Dupré J, 1986. Sex, gender, and essence. Midwest studies in philosophy 11:441-457.

Eppley M, Lee A, Dellinger R, Swank A, 2026. There is No Consensus on Biological Sex. Ecology Letters 29:e70350.

Evron A, 2024. What do sexes have to do with (models of) sexual selection? Philosophy of Science 91:310-328.

Frederick DA, 2025. Gamete Size Is Essential for Understanding Sex and Sexual Selection in Humans, Animals, Plants, and Algae But Configurations of Sex-Linked Traits Are Practically Important for Understanding Sex. Arch Sex Behav:1-5.

Gorelick R, Carpinone J, Derraugh LJ, 2013. Fundamental differences between females and males? Challenging popular myths of sex, gender and biology: Springer. p. 9-22.

Gorelick R, Carpinone J, Derraugh LJ, 2017. No universal differences between female and male eukaryotes: anisogamy and asymmetrical female meiosis. Biological Journal of the Linnean Society 120:1-21.

Griffiths PE, 2020. Sex is real. Aeon Magazine: September 21st

Griffiths PE, Spencer HG, 2025. Biology should not dispense with sexes. Curr Biol 35:R244-R248. doi: 10.1016/j.cub.2025.02.024.

Lehtonen J, 2021. The legacy of Parker, Baker and Smith 1972: Gamete competition, the evolution of anisogamy, and model robustness. Cells 10:573.

McLaughlin JF, Brock KM, Gates I, Pethkar A, Piattoni M, Rossi A, Lipshutz SE, 2023. Multivariate Models of Animal Sex: Breaking Binaries Leads to a Better Understanding of Ecology and Evolution. Integr Comp Biol 63:891-906. doi: 10.1093/icb/icad027.

Rehmann-Sutter C, Hiort O, Krämer UM, Malich L, Spielmann M, 2023. Is sex still binary? Med Genet 35:173-180. doi: 10.1515/medgen-2023-2039.

Rifkin MJ, Garson J, 2023. Sex by design: a new account of the animal sexes. Biology & Philosophy 38:13.

Schärer L, 2017. The varied ways of being male and female. Molecular reproduction and development 84:94-104.

Smiley KO, Munley KM, Aghi K, Lipshutz SE, Patton TM, Pradhan DS, Solomon-Lane TK, Sun SD, 2024. Sex diversity in the 21st century: Concepts, frameworks, and approaches for the future of neuroendocrinology. Horm Behav 157:23. doi: 10.1016/j.yhbeh.2023.105445.

Staedler YM, Kreisberger T, Manafzadeh S, Chartier M, Handschuh S, Pamperl S, Sontag S, Paun O, Schönenberger J, 2018. Novel computed tomography-based tools reliably quantify plant reproductive investment. Journal of Experimental Botany 69:525-535.

Togashi T, Nomura K, Mochizuki K, Parker GA, Horinouchi Y, 2024. An ulvophycean marine green alga produces large parthenogenetic isogametes as predicted by the gamete dynamics model for the evolution of anisogamy. Biology Letters 20:20240489.

Watkins A, DiMarco M, 2025. Sex eliminativism. Biology & Philosophy 40:2.

Wright CM, 2025. Why there are exactly two sexes. Arch Sex Behav 54:3941-3945. doi: 10.1007/s10508-025-03348-3.
